# 4-(4-Hy­droxy­phen­yl)butan-2-one

**DOI:** 10.1107/S1600536811017272

**Published:** 2011-05-14

**Authors:** Jian-Guo Wang

**Affiliations:** a School of Chemistry & Chemical Engineering, Jiujiang University, Jiujiang 332005, People’s Republic of China

## Abstract

In the title compound, C_10_H_12_O_2_, the substituted benzene ring is inclined at a dihedral angle of 75.9 (1)° to the almost planar butan-2-one substituent (r.m.s. deviation = 0.02 Å). In the crystal, inter­molecular O—H⋯O hydrogen bonds link the mol­ecules into chains along the *a* axis.

## Related literature

For the odour threshold of the title compound, see: Larsen & Poll (1990 ▶); Tang (2006 ▶). For a related structure, see: Kosjek *et al.* (2003 ▶). For the synthesis, see: Smith (1996 ▶).
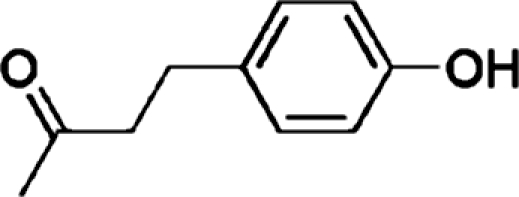


## Experimental

### 

#### Crystal data

C_10_H_12_O_2_
*M* *_r_* = 164.20
Orthorhombic, 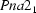

*a* = 14.0242 (13) Å
*b* = 12.4450 (12) Å
*c* = 5.2706 (5) Å
*V* = 919.88 (15) Å^3^
*Z* = 4
Mo *K*α radiation
μ = 0.08 mm^−1^
*T* = 298 K
0.23 × 0.20 × 0.20 mm

#### Data collection

Bruker SMART CCD area-detector diffractometer
5687 measured reflections
1797 independent reflections
1678 reflections with *I* > 2σ(*I*)
*R*_int_ = 0.101

#### Refinement

*R*[*F* ^2^ > 2σ(*F* ^2^)] = 0.064
*wR*(*F* ^2^) = 0.173
*S* = 1.06
1797 reflections
113 parameters
1 restraint
H atoms treated by a mixture of independent and constrained refinement
Δρ_max_ = 0.21 e Å^−3^
Δρ_min_ = −0.15 e Å^−3^

### 

Data collection: *SMART* (Bruker, 1997 ▶); cell refinement: *SAINT* (Bruker, 1997 ▶); data reduction: *SAINT*; program(s) used to solve structure: *SHELXS97* (Sheldrick, 2008 ▶); program(s) used to refine structure: *SHELXL97* (Sheldrick, 2008 ▶); molecular graphics: *SHELXTL* (Sheldrick, 2008 ▶); software used to prepare material for publication: *SHELXTL*.

## Supplementary Material

Crystal structure: contains datablocks I, global. DOI: 10.1107/S1600536811017272/sj5132sup1.cif

Structure factors: contains datablocks I. DOI: 10.1107/S1600536811017272/sj5132Isup2.hkl

Supplementary material file. DOI: 10.1107/S1600536811017272/sj5132Isup3.cml

Additional supplementary materials:  crystallographic information; 3D view; checkCIF report

## Figures and Tables

**Table 1:** Hydrogen-bond geometry (Å, °)

*D*—H⋯*A*	*D*—H	H⋯*A*	*D*⋯*A*	*D*—H⋯*A*
O1—H1⋯O2^i^	0.91 (5)	1.97 (5)	2.842 (4)	161 (5)

Symmetry code: (i) 
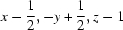
.

**Fig. 1.**
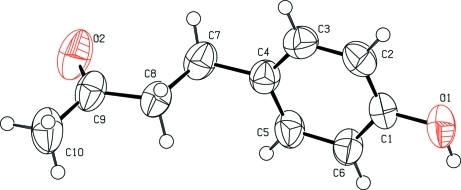
View of the molecule of (I) showing the atom-labelling scheme. Displacement ellipsoids are drawn at the 50% probability level.

**Fig. 2.**
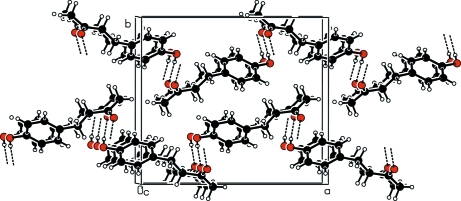
The crystal packing for (I), with O—H···O interactions shown as dashed lines.

## supplementary crystallographic information

### Comment

The title compound, known as raspberry ketone, was originally extracted from raspberry and possesses the flavour of raspberries (Larsen & Poll 1990). However, the content of raspberry ketone in raspberry is very low (Tang, 2006).

The asymmetric unit of (I) contains one independent molecule (Fig. 1). The bond lengths and angles are normal and similar to those in a related structure (Kosjek *et al.*, 2003; Smith, 1996). The hydroxy substituted C1···C6 benzene ring is inclined at a dihedral angle of 75.9 (1)° from the planar C7···C10(O2) butan-2-one substituent (rms deviation 0.02Å). In the crystal structure, a one-dimensional network structure (Fig. 2) is formed by intermolecular O—H···O hydrogen bonds (Table 1).

### Experimental

The title compound was synthesized according to a reported procedure from the corresponding *p*-hydroxybenzaldehyde (Smith, 1996). After recrystallisation from ethanol, the title compound was dissolved in dilute aqeous NaOH. Hydrochloric acid 1:1 (*v*/*v*) was slowly added to adjust to pH = 5. The mixture was left for a week after which colourless block-like crystals were obtained.

### Refinement

All the carbon-bounded hydrogen atoms were located at their ideal positions with the C—H=0.93 Å,*C*—H=0.96 Å, C—H=0.97 Å, and *U*_iso_(H)=1.2*U*_eq_(C). The hydrogen atom bonded to the oxygen atom was located from the difference map and refined with the restraints of O—H = 0.91 (5)Å and *U*_iso_(H)=1.5*U*_eq_(O).

### Crystal data

**Table:**

C_10_H_12_O_2_	*F*(000) = 352
*M**_r_* = 164.20	*D*_x_ = 1.186 Mg m^−^^3^
Orthorhombic, *P**n**a*2_1_	Mo *K*α radiation, λ = 0.71073 Å
Hall symbol: P 2c -2n	Cell parameters from 2580 reflections
*a* = 14.0242 (13) Å	θ = 2.2–24.5°
*b* = 12.4450 (12) Å	µ = 0.08 mm^−^^1^
*c* = 5.2706 (5) Å	*T* = 298 K
*V* = 919.88 (15) Å^3^	Block, colourless
*Z* = 4	0.23 × 0.20 × 0.20 mm

### Data collection

**Table:**

Bruker SMART CCD area-detector diffractometer	1678 reflections with *I* > 2σ(*I*)
Radiation source: fine-focus sealed tube	*R*_int_ = 0.101
graphite	θ_max_ = 26.0°, θ_min_ = 2.2°
φ and ω scans	*h* = −16→17
5687 measured reflections	*k* = −12→15
1797 independent reflections	*l* = −6→6

### Refinement

**Table:**

Refinement on *F*^2^	Primary atom site location: structure-invariant direct methods
Least-squares matrix: full	Secondary atom site location: difference Fourier map
*R*[*F*^2^ > 2σ(*F*^2^)] = 0.064	Hydrogen site location: inferred from neighbouring sites
*wR*(*F*^2^) = 0.173	H atoms treated by a mixture of independent and constrained refinement
*S* = 1.06	*w* = 1/[σ^2^(*F*_o_^2^) + (0.1055*P*)^2^ + 0.082*P*] where *P* = (*F*_o_^2^ + 2*F*_c_^2^)/3
1797 reflections	(Δ/σ)_max_ < 0.001
113 parameters	Δρ_max_ = 0.21 e Å^−^^3^
1 restraint	Δρ_min_ = −0.15 e Å^−^^3^

### Special details

**Table:**

Geometry. All e.s.d.'s (except the e.s.d. in the dihedral angle between two l.s. planes) are estimated using the full covariance matrix. The cell e.s.d.'s are taken into account individually in the estimation of e.s.d.'s in distances, angles and torsion angles; correlations between e.s.d.'s in cell parameters are only used when they are defined by crystal symmetry. An approximate (isotropic) treatment of cell e.s.d.'s is used for estimating e.s.d.'s involving l.s. planes.
Refinement. Refinement of *F*^2^ against ALL reflections. The weighted *R*-factor *wR* and goodness of fit *S* are based on *F*^2^, conventional *R*-factors *R* are based on *F*, with *F* set to zero for negative *F*^2^. The threshold expression of *F*^2^ > σ(*F*^2^) is used only for calculating *R*-factors(gt) *etc*. and is not relevant to the choice of reflections for refinement. *R*-factors based on *F*^2^ are statistically about twice as large as those based on *F*, and *R*- factors based on ALL data will be even larger.

### Fractional atomic coordinates and isotropic or equivalent isotropic displacement parameters (Å^2^)

**Table:**

	*x*	*y*	*z*	*U*_iso_*/*U*_eq_	
C1	0.38120 (16)	0.29404 (19)	0.1522 (5)	0.0521 (6)	
C2	0.38140 (19)	0.3629 (2)	0.3552 (6)	0.0627 (7)	
H2	0.3252	0.3966	0.4048	0.075*	
C3	0.46513 (19)	0.3825 (2)	0.4863 (5)	0.0622 (7)	
H3	0.4644	0.4300	0.6224	0.075*	
C4	0.54959 (17)	0.33317 (19)	0.4198 (5)	0.0532 (6)	
C5	0.54753 (17)	0.2641 (2)	0.2135 (6)	0.0604 (7)	
H5	0.6035	0.2298	0.1648	0.072*	
C6	0.46503 (18)	0.2445 (2)	0.0779 (6)	0.0577 (6)	
H6	0.4658	0.1987	−0.0614	0.069*	
C7	0.6424 (2)	0.3537 (2)	0.5558 (5)	0.0639 (7)	
H7A	0.6750	0.2860	0.5835	0.077*	
H7B	0.6293	0.3854	0.7203	0.077*	
C8	0.70702 (18)	0.4287 (2)	0.4049 (5)	0.0573 (6)	
H8A	0.7130	0.4007	0.2338	0.069*	
H8B	0.6764	0.4984	0.3933	0.069*	
C9	0.80525 (18)	0.4441 (2)	0.5120 (5)	0.0622 (7)	
C10	0.8711 (2)	0.5121 (3)	0.3595 (8)	0.0852 (11)	
H10A	0.9327	0.5135	0.4389	0.128*	
H10B	0.8462	0.5839	0.3495	0.128*	
H10C	0.8768	0.4828	0.1917	0.128*	
O1	0.29729 (13)	0.27805 (18)	0.0238 (5)	0.0713 (6)	
H1	0.303 (3)	0.230 (4)	−0.105 (10)	0.107*	
O2	0.82901 (17)	0.4040 (2)	0.7093 (5)	0.0906 (8)	

### Atomic displacement parameters (Å^2^)

**Table:**

	*U*^11^	*U*^22^	*U*^33^	*U*^12^	*U*^13^	*U*^23^
C1	0.0400 (11)	0.0505 (12)	0.0657 (15)	−0.0032 (9)	−0.0021 (10)	0.0083 (11)
C2	0.0480 (12)	0.0688 (15)	0.0715 (16)	0.0096 (11)	0.0080 (11)	0.0007 (13)
C3	0.0634 (15)	0.0657 (14)	0.0576 (13)	0.0022 (12)	0.0011 (12)	−0.0075 (12)
C4	0.0509 (12)	0.0542 (12)	0.0544 (13)	−0.0026 (10)	−0.0061 (10)	0.0062 (10)
C5	0.0419 (11)	0.0611 (13)	0.0782 (17)	0.0042 (10)	−0.0035 (11)	−0.0084 (13)
C6	0.0481 (13)	0.0537 (13)	0.0714 (15)	−0.0009 (10)	−0.0056 (11)	−0.0101 (11)
C7	0.0652 (16)	0.0635 (14)	0.0629 (16)	−0.0026 (13)	−0.0180 (13)	0.0059 (12)
C8	0.0547 (14)	0.0560 (13)	0.0612 (14)	−0.0002 (10)	−0.0136 (11)	−0.0033 (12)
C9	0.0554 (13)	0.0494 (12)	0.0818 (18)	0.0059 (11)	−0.0183 (14)	−0.0129 (13)
C10	0.0637 (18)	0.0765 (19)	0.115 (3)	−0.0186 (14)	−0.0073 (17)	−0.014 (2)
O1	0.0410 (9)	0.0783 (13)	0.0947 (15)	−0.0026 (8)	−0.0109 (10)	−0.0017 (12)
O2	0.0806 (16)	0.0855 (14)	0.1056 (18)	−0.0036 (12)	−0.0449 (14)	0.0113 (14)

### Geometric parameters (Å, °)

**Table:**

C1—C2	1.371 (4)	C7—C8	1.525 (4)
C1—O1	1.372 (3)	C7—H7A	0.9700
C1—C6	1.384 (4)	C7—H7B	0.9700
C2—C3	1.384 (4)	C8—C9	1.501 (3)
C2—H2	0.9300	C8—H8A	0.9700
C3—C4	1.379 (4)	C8—H8B	0.9700
C3—H3	0.9300	C9—O2	1.200 (4)
C4—C5	1.386 (4)	C9—C10	1.489 (5)
C4—C7	1.508 (3)	C10—H10A	0.9600
C5—C6	1.382 (4)	C10—H10B	0.9600
C5—H5	0.9300	C10—H10C	0.9600
C6—H6	0.9300	O1—H1	0.91 (5)
			
C2—C1—O1	118.5 (2)	C8—C7—H7A	109.3
C2—C1—C6	119.8 (2)	C4—C7—H7B	109.3
O1—C1—C6	121.6 (2)	C8—C7—H7B	109.3
C1—C2—C3	120.1 (2)	H7A—C7—H7B	108.0
C1—C2—H2	120.0	C9—C8—C7	115.3 (2)
C3—C2—H2	120.0	C9—C8—H8A	108.5
C4—C3—C2	121.6 (2)	C7—C8—H8A	108.5
C4—C3—H3	119.2	C9—C8—H8B	108.5
C2—C3—H3	119.2	C7—C8—H8B	108.5
C3—C4—C5	117.2 (2)	H8A—C8—H8B	107.5
C3—C4—C7	123.0 (2)	O2—C9—C10	122.1 (3)
C5—C4—C7	119.7 (2)	O2—C9—C8	121.9 (3)
C6—C5—C4	122.2 (2)	C10—C9—C8	116.0 (3)
C6—C5—H5	118.9	C9—C10—H10A	109.5
C4—C5—H5	118.9	C9—C10—H10B	109.5
C5—C6—C1	119.1 (2)	H10A—C10—H10B	109.5
C5—C6—H6	120.4	C9—C10—H10C	109.5
C1—C6—H6	120.4	H10A—C10—H10C	109.5
C4—C7—C8	111.6 (2)	H10B—C10—H10C	109.5
C4—C7—H7A	109.3	C1—O1—H1	113 (3)
			
O1—C1—C2—C3	−178.8 (3)	C2—C1—C6—C5	1.2 (4)
C6—C1—C2—C3	−0.3 (4)	O1—C1—C6—C5	179.6 (3)
C1—C2—C3—C4	−0.7 (4)	C3—C4—C7—C8	−102.6 (3)
C2—C3—C4—C5	0.9 (4)	C5—C4—C7—C8	75.6 (3)
C2—C3—C4—C7	179.1 (3)	C4—C7—C8—C9	−173.6 (2)
C3—C4—C5—C6	0.0 (4)	C7—C8—C9—O2	−3.5 (4)
C7—C4—C5—C6	−178.3 (3)	C7—C8—C9—C10	176.7 (2)
C4—C5—C6—C1	−1.0 (4)		

### Hydrogen-bond geometry (Å, °)

**Table:**

*D*—H···*A*	*D*—H	H···*A*	*D*···*A*	*D*—H···*A*
O1—H1···O2^i^	0.91 (5)	1.97 (5)	2.842 (4)	161 (5)

Symmetry codes: (i) *x*−1/2, −*y*+1/2, *z*−1.
